# Self-reported and objective sleep duration in patients with CKD: are they telling the same story?

**DOI:** 10.1590/2175-8239-JBN-2022-0015en

**Published:** 2022-08-19

**Authors:** Kalyanna S. Bezerra de Carvalho, Julia C. Lauar, Luciano F. Drager, Rosa M.A. Moyses, Rosilene M. Elias

**Affiliations:** 1 Universidade de São Paulo, Hospital das Clínicas, Departamento de Medicina, Divisão Renal, São Paulo, SP, Brazil; 2 Universidade de São Paulo, Hospital das Clínicas, Instituto do Coração, Departamento de Cardiologia, São Paulo, SP, Brazil; 3 Universidade Nove de Julho, Departamento de Pós-Graduação, São Paulo, SP, Brazil

**Keywords:** Actigraphy, Renal Insufficiency, Chronic, Conservative Treatment, Actigrafia, Insuficiência Renal Crônica, Tratamento Conservador

## Abstract

**Introduction:**

There is disagreement between data on sleep duration obtained from questionnaires and objective measurements. Whether this is also true for individuals with CKD is unknown. Here we compared self-reported sleep duration with sleep duration obtained by actigraphy.

**Methods:**

This prospective study included adult individuals with stage 3 CKD recruited between September/2016 and February/2019. We evaluated subjective sleep duration by asking the following question: “How many hours of actual sleep did you get at night?”

**Results:**

Patients (N=34) were relatively young (51 ± 13 years). Self-reported and measured sleep duration were 7.1 ± 1.7 and 6.9 ± 1.6 hours, respectively, with no correlation between them (p=0.165). Although the mean difference between measurements was 0.21 h, the limits of agreement ranged from -3.7 to 4.1 h.

**Conclusion:**

Patients with CKD who are not on dialysis have an erroneous sleep perception. Data on sleep duration should be preferentially obtained from objective measurements in patients with CKD.

## Resumo

**Introdução:**

Há discordância entre os dados sobre duração do sono obtidos a partir de questionários e medições objetivas. Não se sabe se isto também é verdade para indivíduos com DRC. Aqui comparamos a duração do sono autorrelatada com a duração do sono obtida por meio de actigrafia.

**Métodos:**

Este estudo prospectivo incluiu indivíduos adultos com DRC estadio 3 recrutados entre Setembro/2016 e Fevereiro/2019. Avaliamos a duração subjetiva do sono, fazendo a seguinte questão: “Quantas horas de sono real você teve à noite?”

**Resultados:**

Os pacientes (N=34) eram relativamente jovens (51 ± 13 anos). A duração do sono autorrelatada e mensurada foi de 7,1 ± 1,7 e 6,9 ± 1,6 horas, respectivamente, sem correlação entre elas (p=0,165). Embora a diferença média entre as medições tenha sido de 0,21 h, os limites de concordância variaram de -3,7 a 4,1 h.

**Conclusão:**

Pacientes com DRC que não estão em diálise apresentam uma percepção equivocada do sono. Dados sobre a duração do sono devem ser obtidos preferencialmente a partir de medições objetivas em pacientes com DRC.

## Introduction

Sleep duration is a fundamental concept in epidemiological studies since total sleep time has important effects on health. Observational studies have produced discrepant results on the relationship between sleep duration and several outcomes. Short subjective sleep duration has been associated with hypertension, proteinuria, and higher risk of death in population-based studies^1^. For the general population, subjective sleep duration is already known to be inconsistent with objective measures obtained by actigraphy or polysomnography^2^. Whether this is also true for individuals with CKD is unknown.

Recently, Lee et al.^3^ examined the relationship between sleep duration and mortality and quality of life in individuals with CKD. In the mentioned study, sleep duration was obtained by asking a question “How many hours a day do you usually sleep?”. The authors concluded that long sleep duration was associated with poor quality of life in Korean adults with CKD. As in the general population, data on average sleep duration in CKD patients are usually obtained from questionnaires^3-6^. Few studies have measured sleep duration using actigraphy or a full-night registration^7,8^. In addition, these studies did not compare self-reported and objective measurements of sleep duration. Whether patients with CKD misestimate their sleep duration is unknown.

We evaluated the agreement between measured and self-reported sleep duration in patients with CKD on conservative treatment. We hypothesized that there is no agreement between subjective and objective measurements (actigraphy) of total sleep time.

## Methods

Adult individuals attending the nephrology service at Hospital das Clinicas of Universidade de Sao Paulo were recruited between September/2016 and February/2019. Patients participated in a primary study on the association between sleep disorders and bone biomarkers. Eligibility criteria included an estimated glomerular filtration rate (eGFR) indicating stage 3 CKD, according to the CKD-EPI equation^9^, and ability to give informed consent. Exclusion criteria were previous use of bisphosphonate, steroid, calcium carbonate, or anticonvulsants drugs, patients with polycystic kidney disease, hormone therapy, bedridden or wheelchair-dependent, and body mass index > 35 kg/m^2^. The local Research Ethics Board has approved the protocol (CAPpesq #46565615.5.0000.0068) and written consent was obtained from all patients.

Participants were instructed to wear the Actiwatch on the nondominant wrist for a period of 7 consecutive days and nights during a typical week. During actigraphy, participants kept a sleep diary. Subjects were instructed to press the event-marker button on the device to mark events such as time in and out of bed. The wrist Actiwach recorded data in 30-s epochs, and data were downloaded to a computer. An algorithm was used to calculate sleep duration, as the total time of epochs classified as sleep between bedtime and rising time. The parameter of interest was total sleep time (TST), which is the sum, in minutes, of all sleep epochs between sleep onset and sleep end. We evaluated subjective sleep duration by asking the following question: “How many hours of actual sleep did you get at night?” The wrist actigraphy has been validated against polysomnography, demonstrating a correlation of more than 0.9 for total sleep duration in healthy subjects, and is the instrument of choice for assessment of the sleep-wake cycle for prolonged periods of time^10^.

Data are presented as mean ± SD. We used Spearman coefficient to assess correlation between two variables. A Bland-Altman graph was built to test the agreement between reported and measured sleep duration. Difference of sleep duration between these two methods was plot against the mean for each subject. Analyses were performed using SPSS version 21.0 (SPSS Inc., Chicago, IL). A p value < 0.05 was considered to represent a statistically significant difference.

## Results

**Figure 1:**
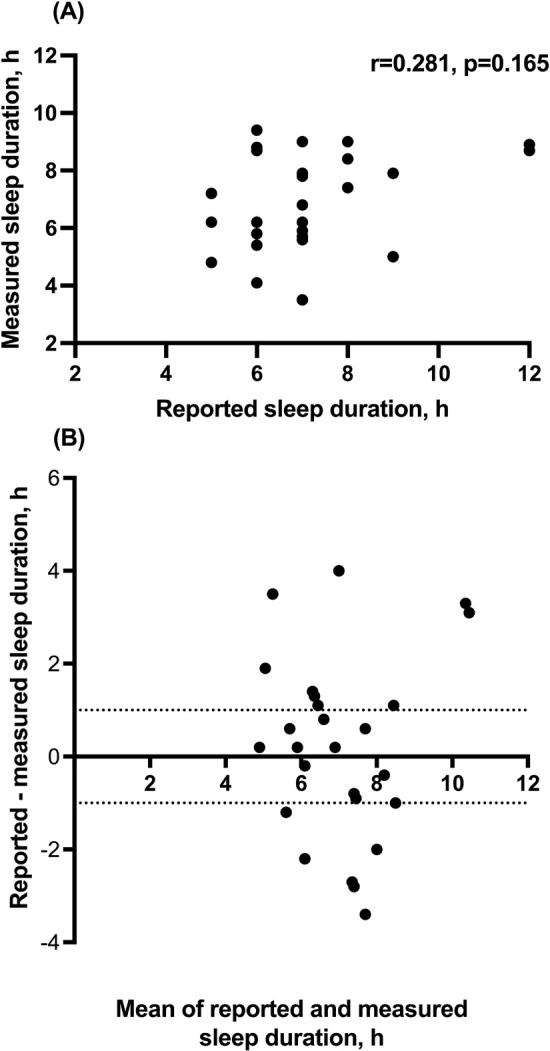
(A) Correlation between self-reported and sleep time measured by actigraphy. (B) Bland-Altman plot of sleep duration reported by patients and measured by actigraphy. Y axis shows the difference between reported and measured sleep duration and the X axis represents the average of these measures. Dotted lines represent -1 and +1 hour difference between measurements.

Patients (N=34) were aged 51 ± 13 years and mostly non-white women. Almost half of the patients were considered overweight (47%). Self-reported sleep duration was 7.1 ± 1.7 hours, varying from 4 to 12 h, whereas measured sleep duration was 6.9 h, varying from 3.5 to 9.4 h. There was no correlation between reported and measured sleep duration (r=0.281, p=0.165), as shown in Figure 1A. The Bland-Altman plot (shown in Figure 1B) shows that although the mean difference between self-reported and measured sleep duration was 0.21 h, limits of agreement ranged from -3.7 to 4.1 h.

As shown in Table 1, using objective sleep duration as a reference, short sleepers (< 6 h) were misclassified in 90% of cases, normal sleep duration (6-8 h) in 28.6%, and long sleepers (> 8 h) in 80% of cases. In general, self-reported sleep duration was underestimated (Table 1). However, in patients reporting a total sleep time between 6-8 h, an equal number of patients slept more or less than the self-reported sleep duration.

**Table 1:** Comparison of self-reported and measured sleep duration according to 3 categories. Gray cells indicate agreement between methods

Self-reported sleep duration	Measured sleep duration		
	< 6 hours	6-8 hours	> 8 hours
< 6 hours	1	3	0
6-8 hours	8	10	8
> 8 hours	1	1	2

Data represent number of patients.

## Discussion

We found no correlation between self-reported and measured sleep duration, supporting former studies with the general population. The discrepancy between subjective and objective sleep duration was so large that most patients were erroneously classified regarding sleep behavior.

In the general population, sleep duration has been associated with several outcomes. However, in the context of CKD, results are contradictory. While some authors have shown an increased risk of mortality and renal function decline associated with short sleep duration^6^, others have shown an improvement of renal function^5^. Yet, long sleep duration has also been associated with higher mortality^3^ and renal function impairment^4,11^. When evaluating CKD incidence, some studies found that a short sleep pattern is a protective factor^12^, whereas other authors have found a similar effect with sleep duration between 6 and 8 hours^13^. Short and/or long sleep has been associated with higher CKD prevalence^7,14,15^, a result that has not been verified by others in a population with coronary artery disease^16^. In most studies in patients with CKD, sleep duration was obtained from questionnaires^3-6,11-13^. Only a few studies measured sleep duration with actigraphy or a full-night registration^7,17^. In addition, these studies did not compare self-reported and measured sleep duration. The method by which sleep duration is obtained is crucial, particularly whether the data were subjectively obtained by questionnaires or objectively measured. If the disagreement between subjective and objective sleep duration is systematic, one can question previous results that have associated sleep duration to outcomes in patients with CKD.

There are several reasons explaining the discrepancies between objective and subjective measurements of sleep duration, namely: 1. Great variability between the methodology used for subjective (a single question, Pittsburgh Sleep Quality Index questionnaire, other questionnaires) and objective sleep measurement (actigraphy, polysomnography); 2. Characteristics of the study population (healthy or with comorbidities, age, gender). There are also some discrepancies between objectively measured sleep and subjective sleep perception, which is reported mainly in patients with insomnia. However, it seems that this is also true for patients with sleep apnea^18^. Since half of our patients were overweight, there is a higher possibility of sleep breathing disorders contributing to the misperception of sleep duration.

In conclusion, patients with CKD not on dialysis misperceive sleep duration. The disagreement between self-reported and measured sleep duration argued for objectively obtained data in this population. Our findings suggest that the interpretation of data from previous studies should be carefully considered depending on how the sleep duration data were collected.
